# Clues to Autistic Behaviors: Exploring the Role of Endocrine Disruptors

**DOI:** 10.1289/ehp.122-A137

**Published:** 2014-05-01

**Authors:** Kellyn S. Betts

**Affiliations:** Kellyn S. Betts writes about environmental contaminants, hazards, and technology for solving environmental problems for publications including *EHP* and *Environmental Science & Technology*.

Two lines of evidence suggest that endocrine disruption may be a factor in autism spectrum disorders (ASDs). First, the observation that males may be four times as likely to be diagnosed with ASDs as females suggests hormonal involvement.^1^ Second, adrenal, gonadal, and thyroid hormones play an important role in fetal neurodevelopment,^2^^,^^3^^,^^4^ and any chemical that interferes with the actions of these hormones therefore has the potential to disrupt brain development. By analyzing samples and data from a prospective birth cohort study, a team of U.S. and Canadian researchers have identified a handful of endocrine-disrupting chemicals (EDCs) they believe merit further study as possible contributors to ASDs.^5^

ASDs encompass a complex set of disorders that have been associated with more than 800 potential genetic risk factors, says Isaac Pessah, associate dean of research and graduate education at the University of California, Davis, who was not involved with the study. In March 2014 the Centers for Disease Control and Prevention revised its estimates of the number of children with ASDs to 1 in 68, up from 1 in 88.^6^

**Figure:**
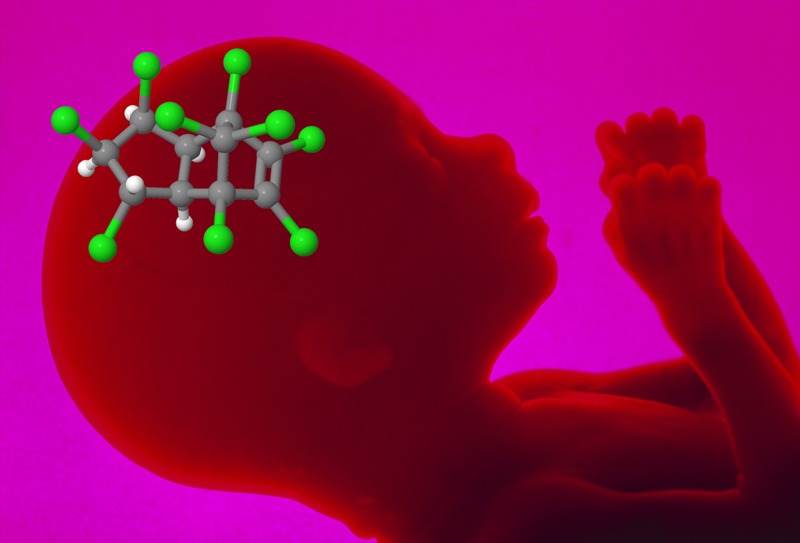
Researchers are investigating endocrine disruption as a possible route by which prenatal chemical exposures may contribute to ASDs. Fetus: © Jellyfish Pictures/Science Source; trans-nonachlor molecule: © Jane Whitney

“We suspect that environment and genetics interact on the fetus prenatally or very early in life to increase or decrease the risk of autism,” says study author Joseph Braun, an epidemiologist at Brown University School of Public Health. Given the importance of identifying environmental risk factors for the conditions, he says surprisingly little research has investigated the role that exposure to EDCs may play in ASDs.

The new study analyzed data for 175 women participating in the Health Outcomes and Measures of the Environment (HOME) Study. The cohort includes women who lived in the Cincinnati, Ohio, metropolitan area when they were pregnant between 2003 and 2006. The women provided blood and urine samples during pregnancy, which were analyzed for 52 endocrine-disrupting chemicals. At ages 4 and 5 years old, the children were rated by their mothers using the Social Responsiveness Scale (SRS), a tool that assesses behaviors typically related to ASDs.

The women’s chemical exposures were similar to levels reported in the 2003–2004 iteration of the National Health and Nutrition Examination Survey. After the researchers adjusted for potential confounding variables, they found that higher maternal exposures to *trans*-nonachlor and PBDE-28 were associated with higher average SRS scores. *Trans*-nonachlor is a component of the highly persistent banned pesticide chlordane, and PBDE-28 is one of the polybrominated diphenyl ether compounds used as flame retardants in commercial goods containing polyurethane foam (including furniture and mattresses) made before 2005.

The study also found negative associations between four additional chemicals and average SRS scores. These included β-hexachlorocyclohexane, an organochlorine pesticide; perfluorooctanoic acid, a compound used to make many industrial polymers and products including Teflon; PCB-178, one of the polychlorinated biphenyls once used in hundreds of industrial and commercial applications; and PBDE-85, another PBDE congener. These results are consistent with some earlier studies but not others; the authors suggest the apparent contradictions may result from differences in the end points studied and the timing of exposure assessment.

“[The new study’s] multi-chemical and multi-outcome approach is innovative and mirrors the real world, where we are all exposed to a mixture of chemicals, and where the neurotoxicants may have different effects that may even depend on the time of exposure,” says Philippe Grandjean, an adjunct professor of environmental health at the Harvard School of Public Health, who was not involved with the study.

But Grandjean also cautions against comparing apples and oranges. “Those substances that have been measured with large imprecision, e.g., because of common short-term variability, are likely to be underestimated as possible contributors to autism-like behaviors,” he explains. On the other hand, “persistent substances will have less imprecision and could therefore erroneously appear as if they are more important.”

Although the study measured only 52 of the many thousands of environmental chemicals to which participants may have been exposed, it is important “because it represents a systematic search for potentially preventable environmental causes of autism,” says Philip Landrigan, professor and chair of preventive medicine in the Icahn School of Medicine at Mount Sinai. He says the study’s approach “falls precisely in line” with the strategy for discovering environmental causes of autism and other neurodevelopmental disabilities that he and other experts outlined in a 2012 *EHP* editorial.^7^
